# Field-based high-throughput phenotyping of plant height in sorghum using different sensing technologies

**DOI:** 10.1186/s13007-018-0324-5

**Published:** 2018-07-04

**Authors:** Xu Wang, Daljit Singh, Sandeep Marla, Geoffrey Morris, Jesse Poland

**Affiliations:** 1 Department of Plant Pathology, 4024 Throckmorton Plant Sciences Center, Kansas State University, Manhattan, KS 66506 USA; 2 Department of Agronomy, 2004 Throckmorton Plant Sciences Center, Kansas State University, Manhattan, KS 66506 USA

**Keywords:** Plant height, Sorghum, Ultrasonic sensor, Laser rangefinder, Kinect time-of-flight camera, Photogrammetry, Digital elevation model

## Abstract

**Background:**

Plant height is an important morphological and developmental phenotype that directly indicates overall plant growth and is widely predictive of final grain yield and biomass. Currently, manually measuring plant height is laborious and has become a bottleneck for genetics and breeding programs. The goal of this research was to evaluate the performance of five different sensing technologies for field-based high throughput plant phenotyping (HTPP) of sorghum [*Sorghum bicolor* (L.) Moench] height. With this purpose, (1) an ultrasonic sensor, (2) a LIDAR-Lite v2 sensor, (3) a Kinect v2 camera, (4) an imaging array of four high-resolution cameras were evaluated on a ground vehicle platform, and (5) a digital camera was evaluated on an unmanned aerial vehicle platform to obtain the performance baselines to measure the plant height in the field. Plot-level height was extracted by averaging different percentiles of elevation observations within each plot. Measurements were taken on 80 single-row plots of a US × Chinese sorghum recombinant inbred line population. The performance of each sensing technology was also qualitatively evaluated through comparison of device cost, measurement resolution, and ease and efficiency of data analysis.

**Results:**

We found the heights measured by the ultrasonic sensor, the LIDAR-Lite v2 sensor, the Kinect v2 camera, and the imaging array had high correlation with the manual measurements (*r* ≥ 0.90), while the heights measured by remote imaging had good, but relatively lower correlation to the manual measurements (*r* = 0.73).

**Conclusion:**

These results confirmed the ability of the proposed methodologies for accurate and efficient HTPP of plant height and can be extended to a range of crops. The evaluation approach discussed here can guide the field-based HTPP research in general.

## Background

The stress from a growing world population and the less favorable environment from the changing climate require substantial improvements of grain crops production by 2050 [1]. To meet this requirement, crop cultivars that have high yield and stress tolerance need to be selected by innovative crop breeding methods. During the past decades spectacular advances in “next generation” DNA sequencing are rapidly reducing the cost of genotyping and enabling genomics-assisted breeding. In contrast, methods for rapid characterization of plant traits have advanced little [2]. Therefore, high-throughput plant phenotyping (HTPP) platforms are needed that can accurately characterize plant phenotypes of large populations in the field with a fraction of the time and labor of manual phenotyping methods [3].

Among various traits, plant height is a fundamental morphological phenotype that directly indicates the plant growth and is highly predictive of biomass and final grain yield. Continuous plant height measurements throughout the season can contribute to identifying different growing stages and consequently select genotypes that have the longer grain filling period to produce more yield. Theoretically, plant height is defined as the shortest distance between the upper boundary (the highest point) of the main photosynthetic tissues (excluding inflorescences) and the ground level [4]. Though easily defined, human bias on identifying the optimal upper boundary selection will potentially exist during manual plant height measurement in the field condition. Also, as plant height is conventionally measured using measuring sticks in the field, this manual data collection is laborious and time-consuming, hence not scalable for large field experiments or many repeated measures at high temporal resolution. Due to these major challenges, proximal sensing technologies become a practical solution to implement HTPP of plant height.

In recent 5 years, various automatic sensing techniques have been explored to measure plant phenotypes under field conditions. Plant height as a quantitative trait can be measured by both sensors and imaging devices and there is a broad range in the mechanics and functional operation of these different sensors and cameras [5]. These examples include using time-of-flight (ToF) techniques where range sensors can directly provide the distance between the sensor and the target object (i.e., the plant canopy). Accordingly, the plant height can be calculated. These sensors typically include the ultrasonic sensor, the light detection and ranging (LiDAR), and the ToF camera. The ultrasonic sensor is a range detector, which can provide measurements that are well correlated with the ground truth measurements if finely tuned [6, 7]. An advantage of the ultrasonic sensor is it senses objects within its operating sound cone and provides an averaged distance which results in an inherent noise filtering of the variable plants within a plot [6]. However, the sensor field of view (FoV) selection is complicated, because narrow FoV may not cover a sufficient area of the plant canopy, while wide FoV may include non-plant objects outside the canopy area.

Compared to the ultrasonic sensor, LiDAR can provide a much higher resolution of the 3D canopy structure when mounted on a mobile field vehicle [8, 9], and the plant height can be extracted through post-processing. However, the digital point cloud of the canopy is very sensitive to LiDAR’s resolution, sampling frequency, and its geo-locations during movement. In addition, LiDAR is considerably more costly than the ultrasonic sensor.

A final option is ToF cameras that can extract distance in image arrays. As inexpensive hardware, a consumer-grade ToF camera, Microsoft Kinect, was developed for simultaneous color and distance detection in 2010. Its upgraded version—Kinect for Windows v2 was released in 2013 and has been proved as a promising tool for 3-dimensional (3D) plant height measurement recently [10, 11]. However, Kinect v2 is sensitive to direct sunlight and requires proper shading to provide suitable measurements [10].

Unlike being measured directly by the ToF techniques, plant height can also be extracted from 3D plant architecture generated by photogrammetry, which uses structure-from-motion (SfM) algorithm to construct 3D digital elevation model (DEM) from common features in 2-dimensional (2D) images [12]. Nguyen et al. [13] designed a field robot with 16 high-resolution cameras on an arc-shaped structure to collect 2D plant images in different viewing angles for 3D plant structure reconstruction. Due to the proximal and limited views of plants from each camera, images have to be taken from multiple cameras or taken at a fast frequency on a mobile platform to obtain sufficient overlaps for photogrammetry. Also, the huge image set requires a high computing capacity for 3D reconstruction. In addition to the ground-based image acquisition, plant images can also be captured from varying viewing angles by a camera carried by an aerial vehicle [14, 15]. Due to the high camera elevation above the ground, much fewer images are needed to generate the DEM of plants for plant height extraction than the images captured by the ground-based platform. However, the geospatial resolution of DEMs from remote imaging is lower than the 3D plant model from proximal imaging.

Through reviewing different sensing technologies for plant height measurement, we could find advantages and drawbacks of each method. Most research focussed on the performance of using only one sensing method for plant height measurement, yet lacked quantitative comparison among other sensing technologies applied on the same plant. Therefore, it is not directly informative for appropriate sensor selection. As such, the overall objective of this work was to evaluate the performance baselines of multiple sensing and imaging technologies for in-field plant height measurement. In this study, we used sorghum as the target crop for phenotyping. As a major grain in the United States and Sub-Sahara Africa, sorghum is one of the most important sources of food, feed, and bioenergy. Field studies of stress tolerance in diverse sorghum germplasm are particularly challenging for phenotyping because of the wide variation in stand establishment and plant morphology. We used two sensors and three types of cameras for sorghum height measurement. A ground vehicle and an unmanned aerial vehicle (UAV) were used as the proximal and the remote sensing platform respectively. We proposed data processing methods for the dataset of each sensing technology, quantitatively compared the results to the manual measurements, discussed the issues with each sensing method, and provided our recommended solutions for field-based high-throughput phenotyping of plant height in sorghum.

## Methods

### Ground-based data acquisition system

**Fig. 1:**
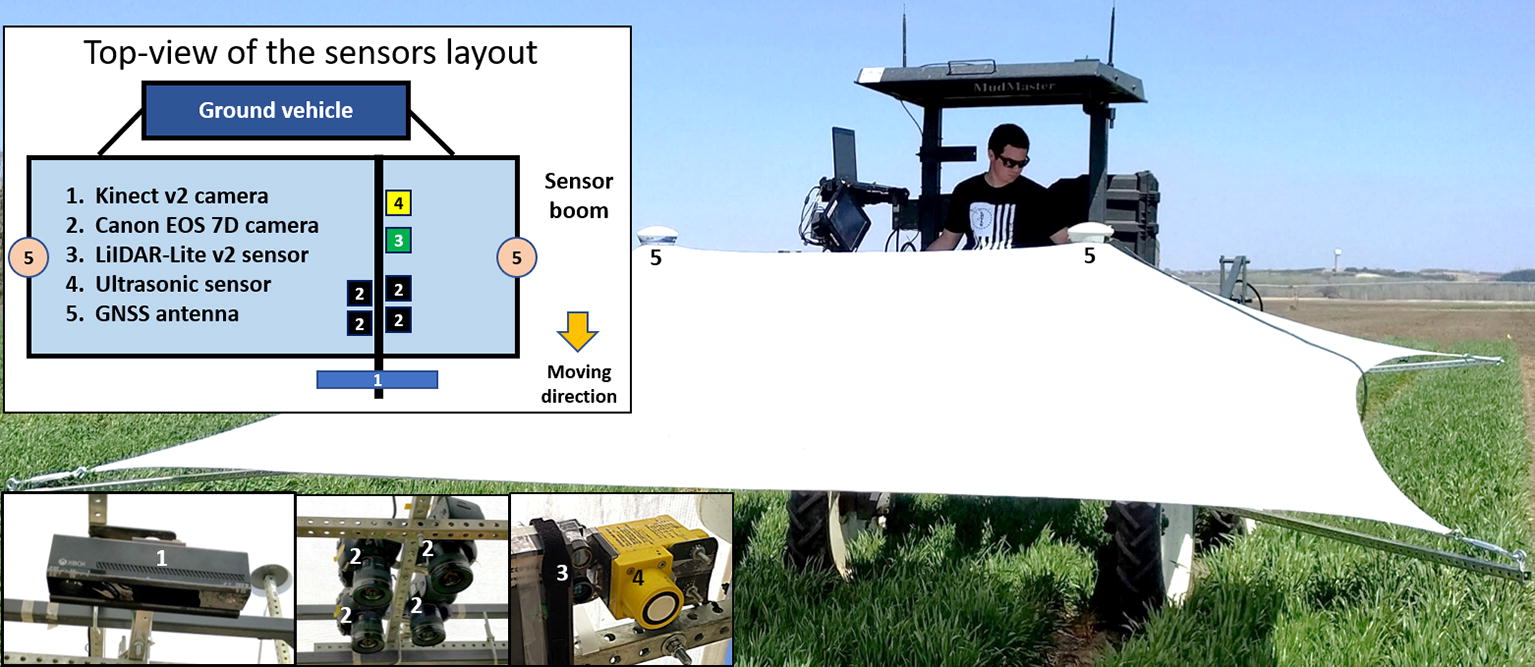
A ground-based HTPP platform on a retrofitted MudMaster sprayer. Sensors mounted on the boom were marked as (*1*) a Kinect for Windows v2, (*2*) four DSLR cameras, (*3*) a LIDAR-Lite v2 sensor, and (*4*) an ultrasonic sensor

A phenotyping mobile unit (PheMU) [16] was developed to collect plant phenotypic data at Kansas State University (KSU), Manhattan, KA. The PheMU equipment (Fig. 1) was retrofitted with a high-clearance sprayer (Bowman Mudmaster, Bowman Manufacturing Co., Inc., Newport, AR, USA). The height of the sensor boom was adjustable to collect sensor measurements of plant height in different stages. To reduce the shadows on the canopy and capture images in a balanced light condition, a rectangle-shape shade sail (Kookaburra OL0131REC, Awnings-USA, Camanche, IA, USA) was mounted on the sensor boom. For plant height measurement, four types of sensing and imaging devices were installed on the boom for simultaneous data collection from a single-row of the plant. The devices included an ultrasonic sensor (U-GAGE Q45U, Banner Engineering Corp., Minneapolis, MN, USA), a LIDAR-Lite v2 (LL2) sensor (LIDAR-Lite v2, Garmin International, Inc., Olathe, KA, USA), a Kinect camera (Kinect for Windows version 2, Microsoft Corp., Redmond, WA, USA), and an imaging array comprised of four digital single-lens reflex (DSLR) cameras (EOS 7D, Canon, Inc., Tokyo, Japan), each with a fixed zoom lens (EF 20 mm f/2.8 USM, Canon, Inc., Tokyo, Japan). All devices were placed in a nadir view of the plant. In addition to the sensing and imaging devices, two GNSS antennae (AG25, Trimble, Westminster, CO, USA) were installed at each end of the sensor boom and connected to two RTK GNSS receivers (FmX integrated display, Trimble, Westminster, CO, USA) for georeferencing the sensor observations and images. An open-source software toolkit [17] for controlling sensors and logging data was developed and deployed on a laptop computer. Raw sensor observations and image file names were attached with time stamps during data collection and were saved as text files on the laptop. Image files from the Kinect and four cameras were transferred to the laptop right after captured.

### Aerial-based image acquisition system

**Fig. 2:**
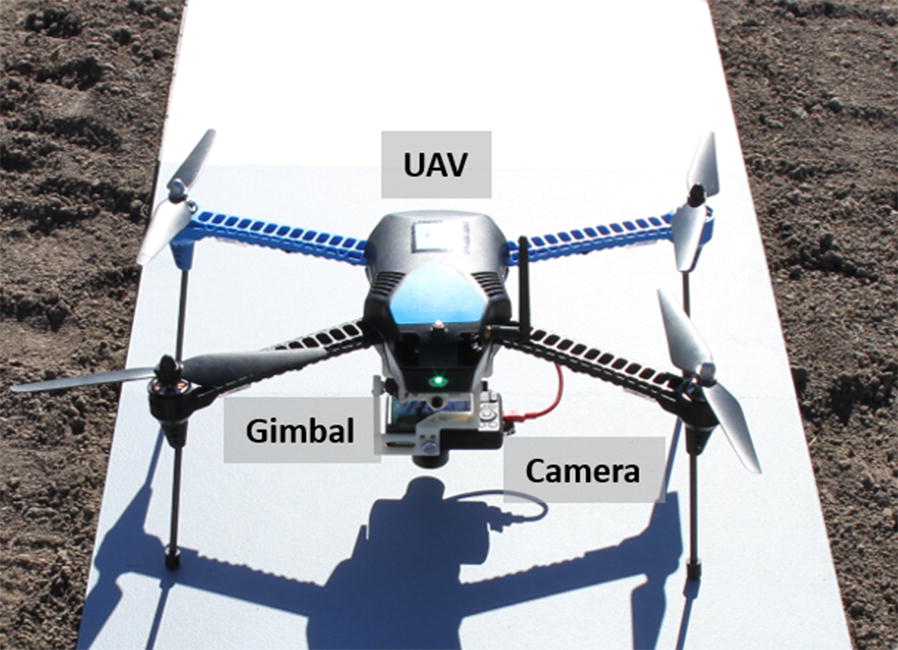
A UAS HTPP platform consisting of an IRIS+ quadcopter, a gimbal, and a digital camera

A low-cost unmanned aerial system (UAS) [15] was integrated for high throughput phenotyping of large breeding nurseries at KSU, Manhattan, KA (Fig. 2). The UAS consisted of a low-cost UAV (IRIS+, 3D Robotics Inc., Berkeley, CA, USA), a custom-designed gimbal (designed by Dr. Dale Schinstock at KSU), and a modified Canon S100 camera (Blue-Green-NIR, 400–760 nm, MaxMax.com LDP LLC, Carlstadt, NJ, USA). The IRIS+ is a light-weight quadcopter UAV, controlled by an open-source Pixhawk autopilot system (PixHawk sponsored by 3D Robotics, www.planner.ardupilot.com). A uBlox GPS with integrated magnetometer was equipped with the IRIS+ for navigation. The gimbal compensated for the UAV movement in the pitch and roll directions during the flight to allow for nadir image acquisition. The GPS inside the S100 camera attached the geo-location data to the raw images for georeferencing.

### Field experiment and data collection

In this study, we used US × Chinese recombinant inbred lines (*n *= 670) generated by crossing chilling-sensitive US line BTx623 with chilling-tolerant Chinese accessions: “Hong Ke Zi”, “Kaoliang”, and “Niu Sheng Zui”. These RILs were replicated twice in the early-season chilling stress experiment, which consisted of 48 rows and 40 ranges in the field. These RILs were planted on April 7th, 2016, around 45 days earlier than the conventional planting date for sorghum, at the KSU Ashland Bottoms Research Farm (39.139 N, 96.619 W) in Manhattan, KA. Despite early planting on April 7th, sorghum seedlings did not emerge until April 28th due to severe drought. Among the 48 rows, phenotypic data were collected on two rows (from the center of the field) across 40 ranges on June 16th and 17th (approximately 50 days after emergence of sorghum seedlings). Overall, 80 single-row plots were traversed. In these 80 plots, two plots were not planted for placing the ground control points (GCPs). The GCPs were used for geospatial correction and the alignment of aerial images. There were 47 plots consisted of BTx623 × Hong Ke Zi RILs, 21 plots of BTx623 × Kaoliang RILs, 7 plots of commercial sorghum Pioneer84G62, and one plot each of Hong Ke Zi, Kaoliang, and BTx623 accessions.

Data was collected by the PheMU on two selected rows sequentially, as the sensor sets could scan only one row for every pass. The sensors and cameras were aligned with the central line of each plot. The Kinect collected three images by every trigger—RGB, depth, and infrared images, although only the depth images were used for plant height extraction. All four DSLR cameras used the same settings listed in Table 1.

**Table 1:** Camera settings

Camera	Resolution (MP)	Image format	Shutter speed	Aperture	ISO	Trigger control
Canon EOS 7D	18	mid-JPG	1/500 s	f/5	400	Canon EDSDK
Canon S100	12	RAW	Shutter priority mode	Auto	Auto	Canon CHDK

Aerial image acquisition using the UAS followed the methodologies of Haghighattalab et al. [15]. Detailed settings of the Canon S100 camera are listed in Table 1. Additional data collection information is shown in Table 2.

**Table 2:** Data collection information

Measuring approach	Carrier	Moving speed (m/s)	Elevation above ground (m)	Sampling rate (Hz)	Collection date (days after emergence)
Ultrasonic sensor	PheMU	0.2	1.35	10	50
LIDAR-Lite v2				5	
DSLR camera array				5	
Kinect v2				1	
Canon S100	IRIS+	2	25	0.33	51
Manual measurement					51

For manual measurements, a barcoded height stick with the 1-cm resolution was used with a barcode scanner (Symbol CS3000, Motorola Inc., Chicago, IL, USA). The manual measurements were scanned into a tablet computer using the Field Book app [18]. In this study, the sorghum height was manually measured from the ground level to the youngest, completely unfurled leaf. Three individual plants in each plot were measured, and the averaged height was used as the plot-level plant height.

### Data processing for height measurement

#### Ultrasonic and LIDAR-Lite v2 point observations

The sensor measurements were georeferenced following the approach of Wang et al. [19]. The boundary coordinates of each field plot were delineated in quantum GIS (QGIS) (www.qgis.org) from the patterns reflected from the sensor measurements [19]. Measurements inside each plot boundary were geotagged with the designated plot identifier. The plant height was calculated as the difference between the sensor elevation above ground and the sensor observation. Two approaches for extracting the plot-level plant height were implemented: (1) the maximum measurement for a given plot, and (2) the average of the top 5% of all measurements inside a given plot. Both plot-level plant height values were compared with the manual measurements.

#### Kinect v2 depth images

**Fig. 3:**
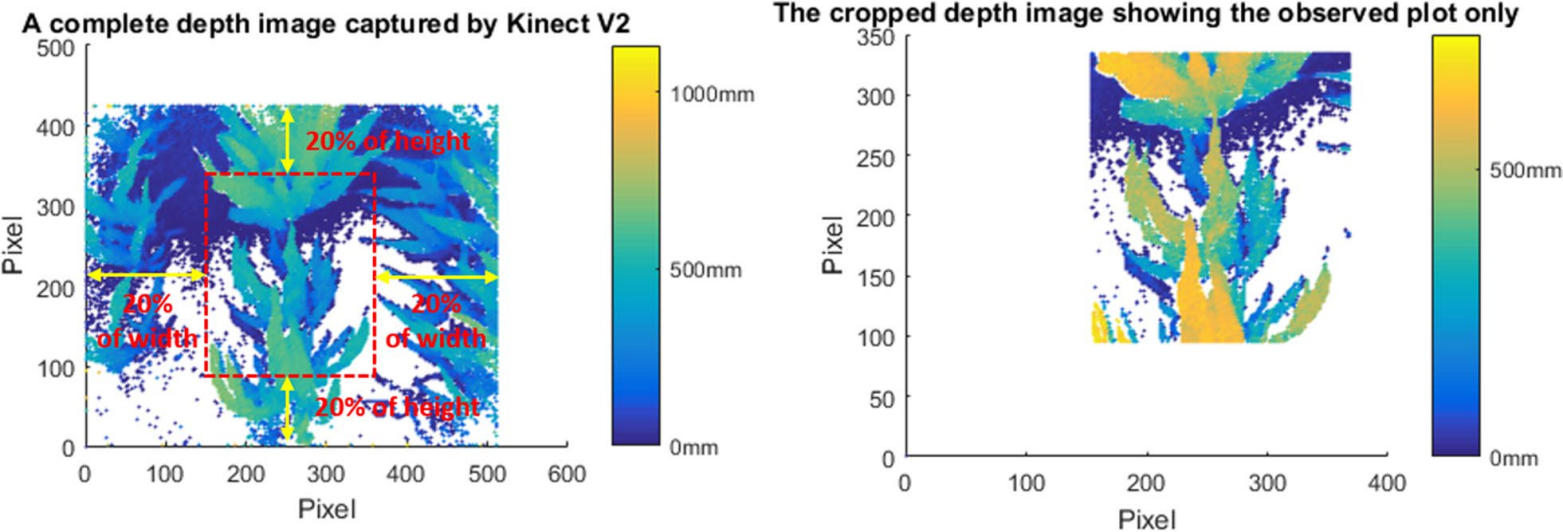
Depth image generated from the Kinect binary output. The left panel shows a complete depth image of one shot. The right panel is the cropped depth image showing the observed plot

The depth images were geotagged and georeferenced following the same approach as for processing the sensor observations to assign plot identifier for each image. As the Kinect camera has a wide FoV, the depth images also contained parts of the neighborhood plots beside the target plot. Therefore, each depth image was cropped by 40% in the width and the height (Fig. 3), leaving only observations for the target plot. Identical to the previous data processing method, the maximum measurement and the average of the top 5% of all the height values from all cropped depth images inside each plot were used as two assessments of plot-level plant height.

#### DSLR camera images

**Fig. 4:**
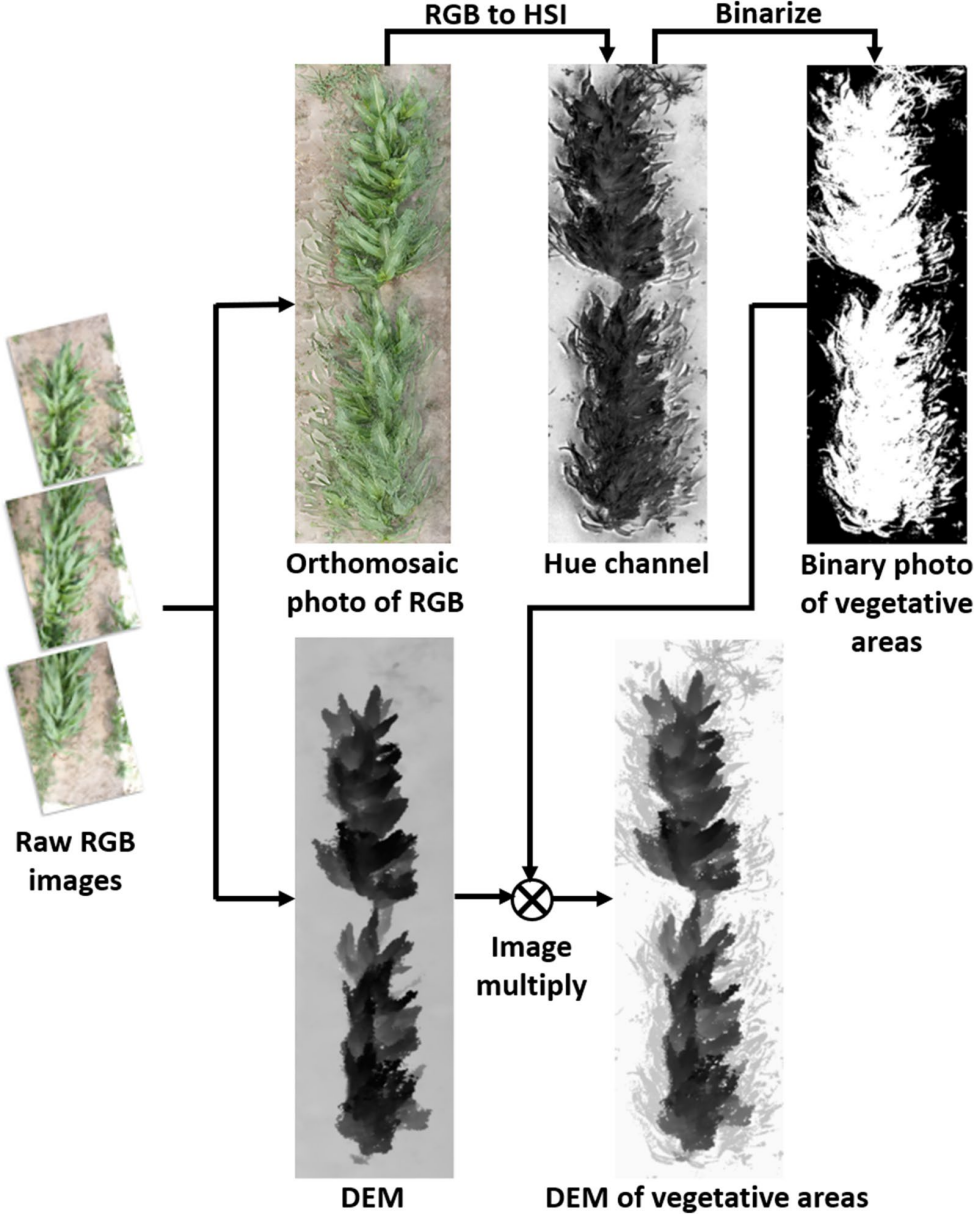
Image processing procedures to generate the proximal DEM from DSLR camera images

The RGB images of DSLR cameras (in the mid-JPEG format) were georeferenced and geotagged following the same approach as to processing the depth images. Images within the same plot were imported to Agisoft PhotoScan (AgiSoft LLC., St. Petersburg, Russia) to generate the red–green–blue (RGB) orthomosaic photo and the DEM of each individual plot. The RGB orthomosaic photo was then converted to the Hue-Saturation-Intensity (HIS) orthomosaic photo, and areas of bare soil were masked using the Hue channel, leaving the vegetative area inside each plot (Fig. 4). The maximum and the averaged top 5% of the elevation values from the DEM within the vegetative area were used as the canopy elevation. Bottom 1% of the elevation values from the DEM within the whole plot area were used as the plot terrain elevation. The plot-level plant height was calculated as the difference between the canopy elevation and the terrain elevation.

#### Aerial images

**Fig. 5:**
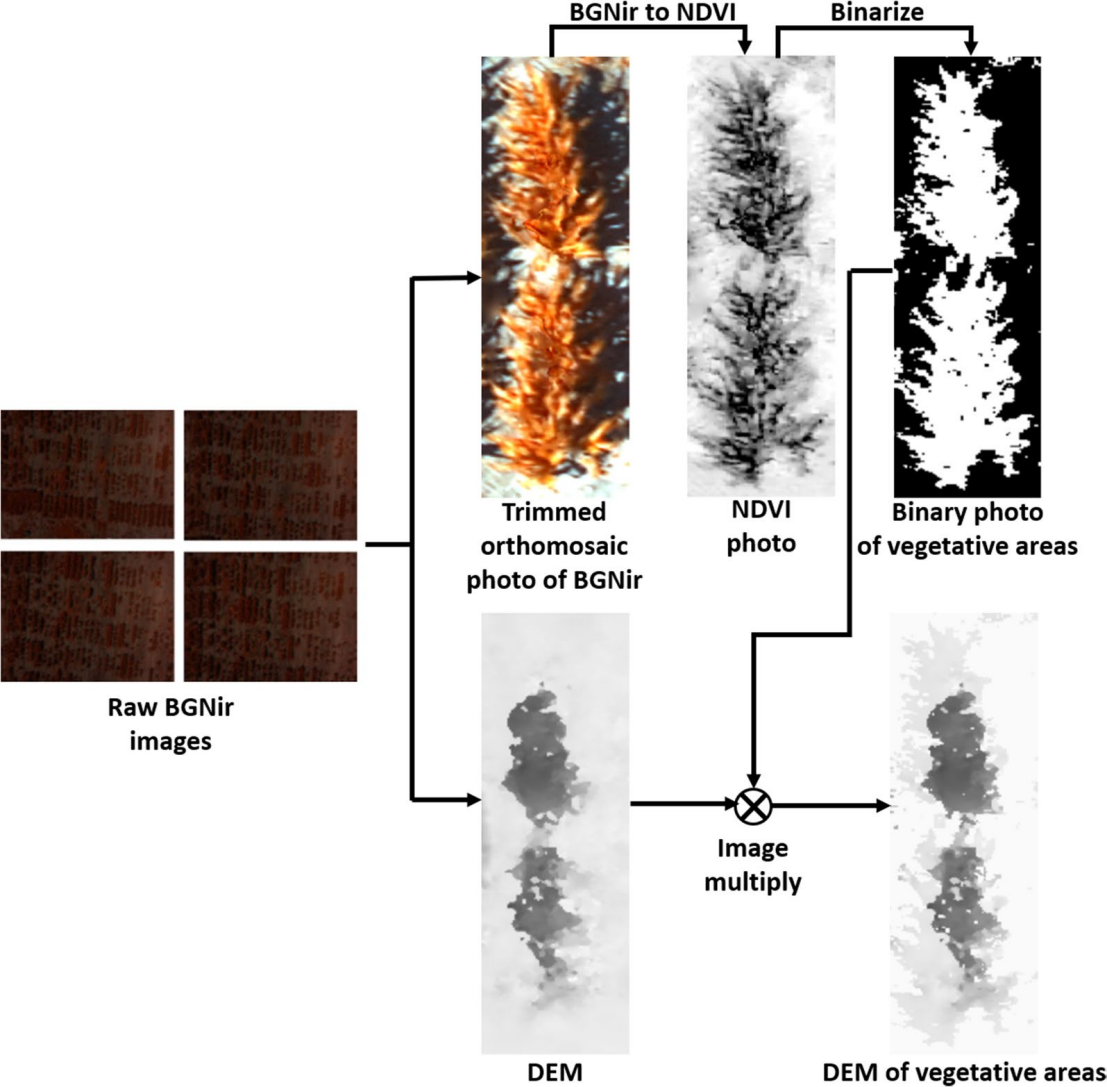
Image processing procedures to generate the remote DEM from aerial images

The raw images (in the CR2 format) of the entire field captured by the Canon S100 were converted to 16-bit linear TIFF images and then corrected for lens distortion using the Canon Digital Photo Professional software. The TIFF images were then imported to Agisoft PhotoScan to generate the blue-green-near infrared (BGNir), orthomosaic photo and the DEM of the entire field. Geo-location information of the GCPs deployed in the field was used by Agisoft Photoscan to optimize the camera locations, resulting in a more accurate DEM. Once generated, the orthomosaic photos and the DEMs of the 80 selected plots were trimmed out by the field plot boundaries in QGIS. Then the BGNir orthomosaic photo was converted to the green normalized difference vegetation index (GNDVI) photo to classify plant versus soil and remove the soil component, leaving the vegetative area inside each plot (Fig. 5). Finally, the plot-level plant height was calculated following the same method as to calculate the plant height from the proximal DEM generated by the DSLR camera images.

## Results and discussion

**Fig. 6:**
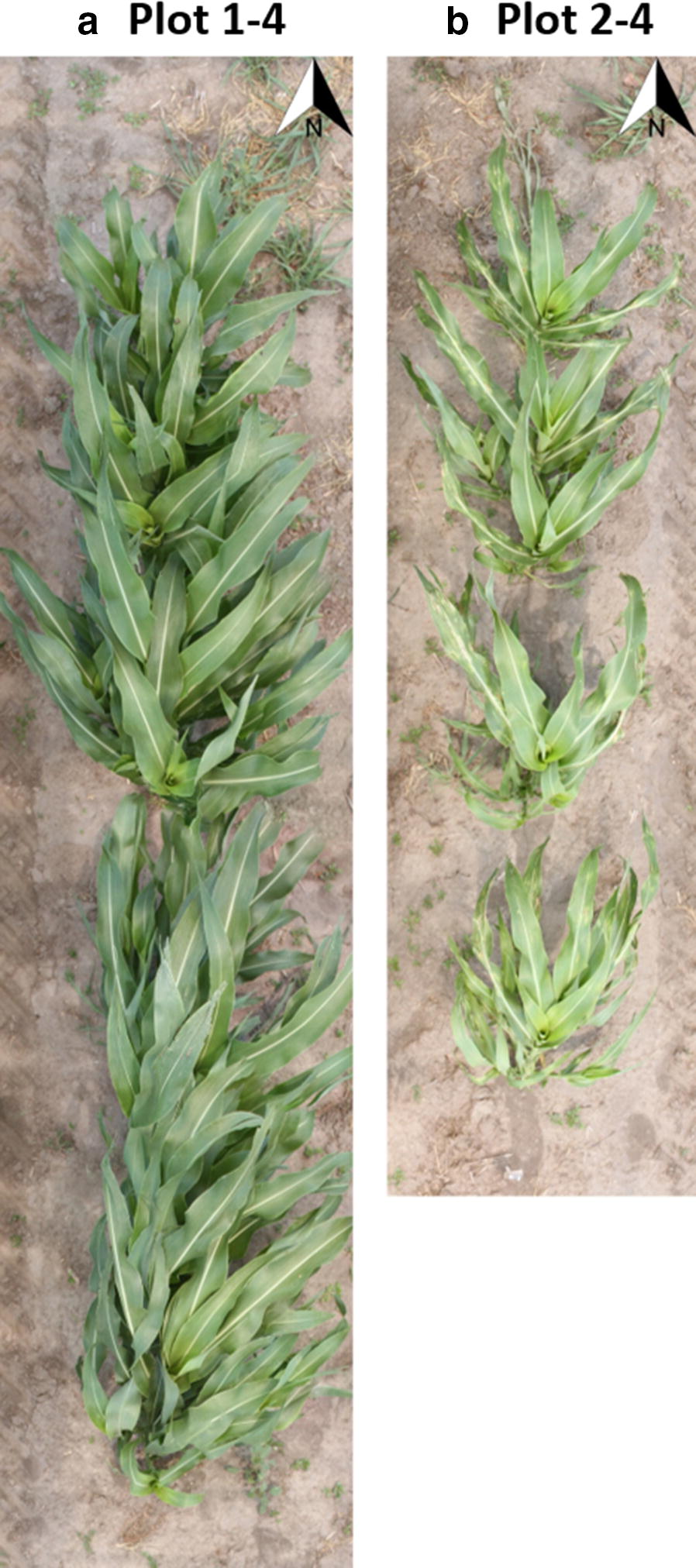
A complete view of **a** a densely vegetative plot and **b** a sparsely vegetative plot

Two plots were selected to demonstrate the height observations and DEMs by each sensing technology. The first plot (Plots 1–4 as shown in Fig. 6a) was a densely vegetative plot with a fully closed canopy, and the second plot (Plots 2–4 as shown in Fig. 6b) was a sparsely vegetative plot with individually observable plants. Due to the early-season chilling stress, the emergence rate of each plot was uneven causing different lengths and plant density for each plot.

### Measurements by the ultrasonic and LIDAR-Lite v2 sensor

**Fig. 7:**
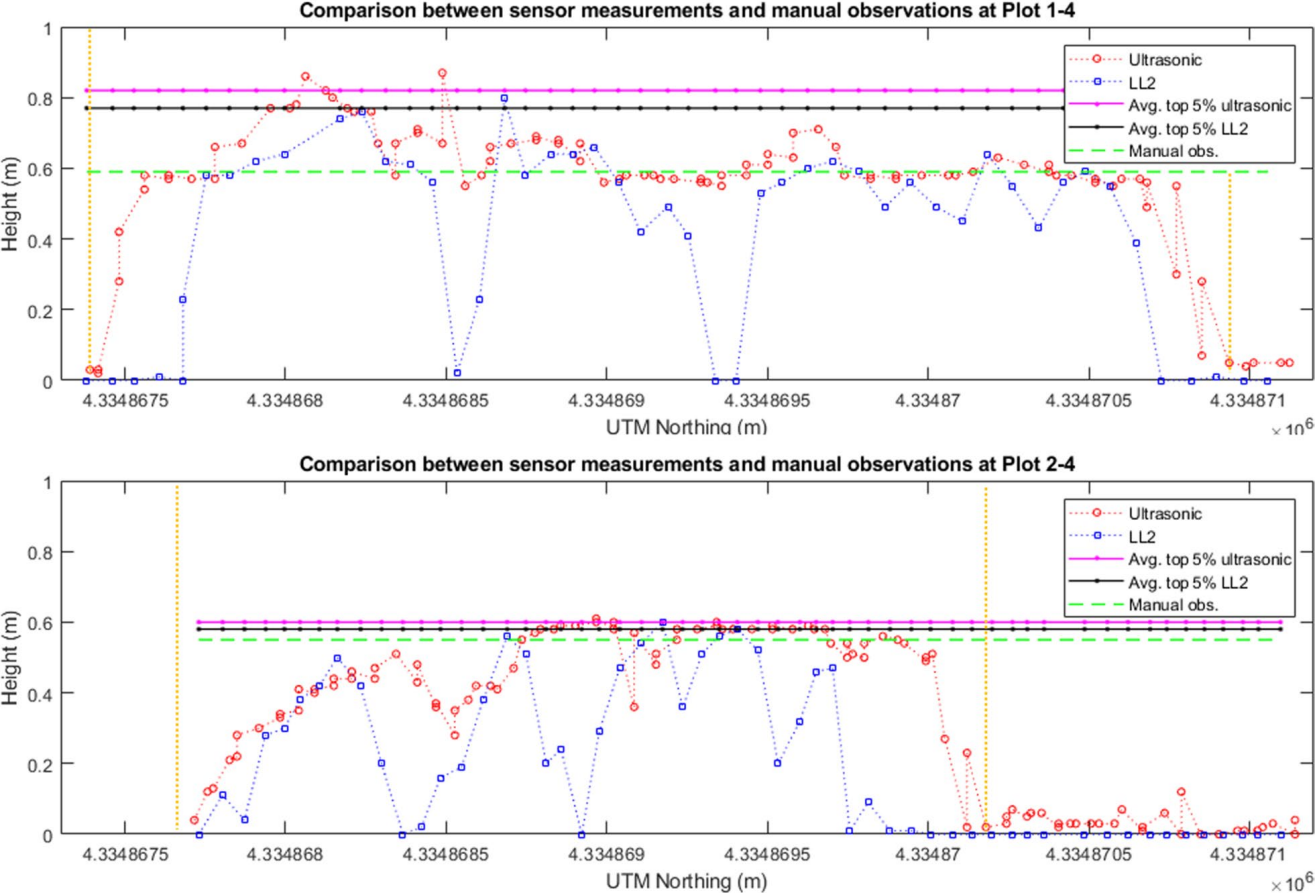
Height observations by the ultrasonic sensor and the LL2 sensor of two plots. The red circles indicate the ultrasonic sensor measurements, while the blue squares indicate the LL2 sensor measurements. The solid pink and black lines indicate the averaged top 5% observations by the ultrasonic sensor and the LIDAR-Lite v2 sensor, respectively. The dashed green line shows the manual (ground truth) observation. The vertical yellow lines mark the start and end of the plot. The *X*-axis in each panel indicate the northing coordinates in meters in the Universal Transverse Mercator (UTM) 14 N coordinate system. The *Y*-axis in each panel indicate the distance measurements in meters

The georeferenced sensor measurements could be considered as a sparse point cloud delineating the profile of the plant canopy (Fig. 7). We observed that the plot-level plant height measured by the ultrasonic sensor was higher than the LL2 sensor. This is likely due to the sampling rate of the ultrasonic sensor being two times higher than the LL2 sensor, resulting in a higher spatial resolution. A second factor was that the ultrasonic sensor has a larger FoV from a sound cone than the FoV from a light pointer of the LL2 sensor. In that case, the light pointer is possible to measure the distance from the sensor to ground between individual leaves while the ultrasonic sensor averaged observations within its operating sound cone. Due to this difference in FoV, the ultrasonic sensor measurements reflected less variance even within the sparsely vegetative plot as the measurements shown in Plots 2–4 (Fig. 7). The ultrasonic sensor, therefore, appears to work with delineating the overall canopy profile, while the LL2 sensor gives an assessment of more peak values of the canopy. In this study, we chose the LL2 sensor because it was a very low-cost range finder originally designed for measuring the UAV elevation above the ground. From the results the LL2 sensor provided, it could be further evaluated whether a LiDAR with a higher sampling rate and a potential 3D point cloud of the canopy structure can be used for extracting more accurate plant height.

### Depth images captured by the Kinect camera

**Fig. 8:**
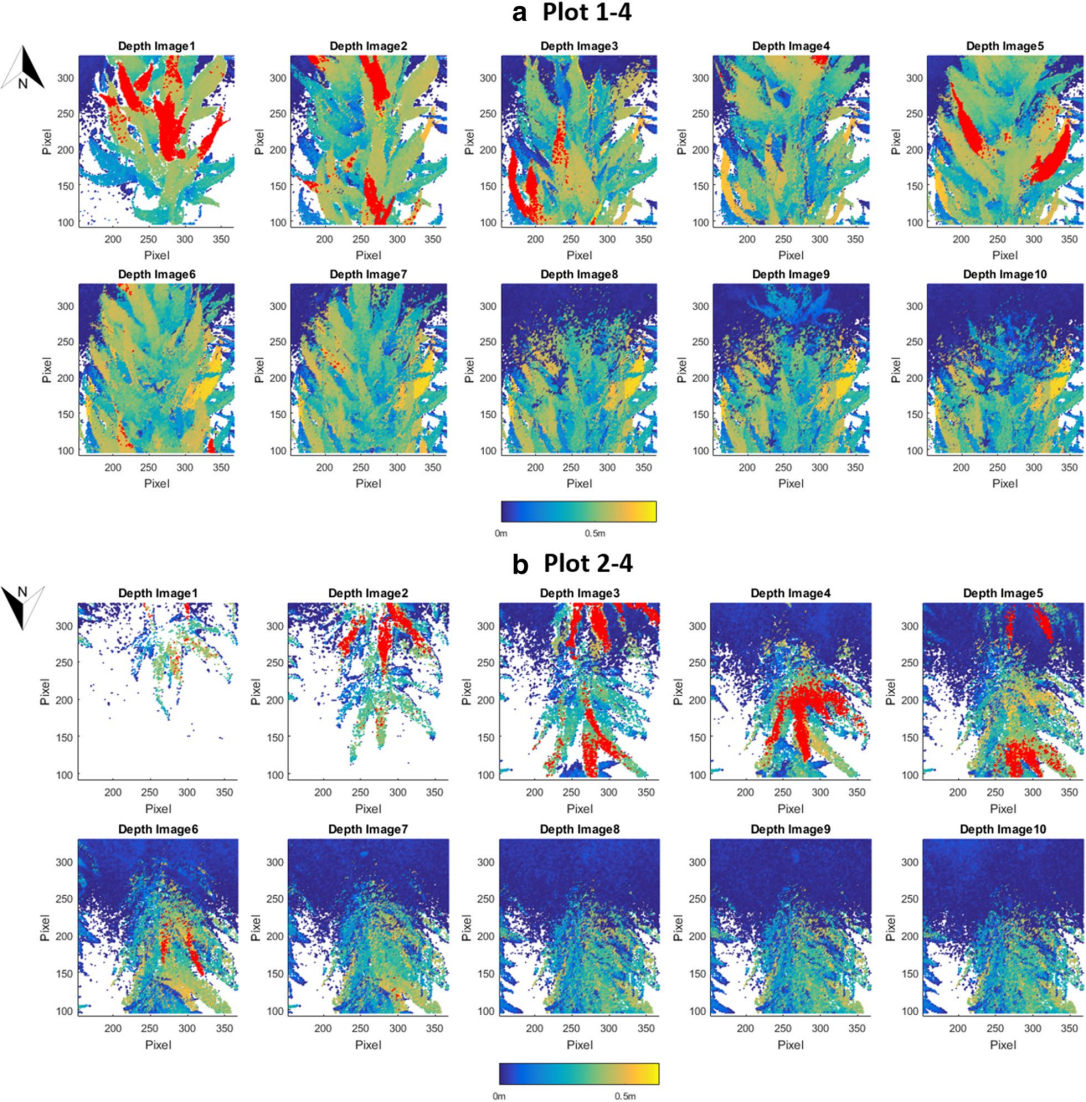
Depth images of two plots. The areas marked in red indicate the top 5% of the canopy height measurements. The upper panel (**a**) shows the depth images of Plot 1–4, while the lower panel (**b**) shows the depth images of Plot 2–4

Each trimmed depth image had around 51K pixels. It could be considered as a 2D projection of a 3D dense point cloud to demonstrate the canopy profile (Fig. 8). The red areas indicate the top 5% of the canopy used to calculate the plot-level plant height. The ten images of Plots 1–4 were collected as the PheMU traversed over the plot from south to north, while the other ten images of Plots 2–4 were collected from north to south. Different from the approach of Jiang et al. [10], we did not generate the plot panorama by stitching all the depth images within each plot because we wanted to include all the depth measurements without downsampling. From the red-colored areas in different depths images, the regions mainly contributing to the plot-level height could be located. Compared with the sensor measurements, 2D depth images highly increase the spatial resolution of the canopy height.

### DEMs generated by proximal and remote imaging

**Fig. 9:**
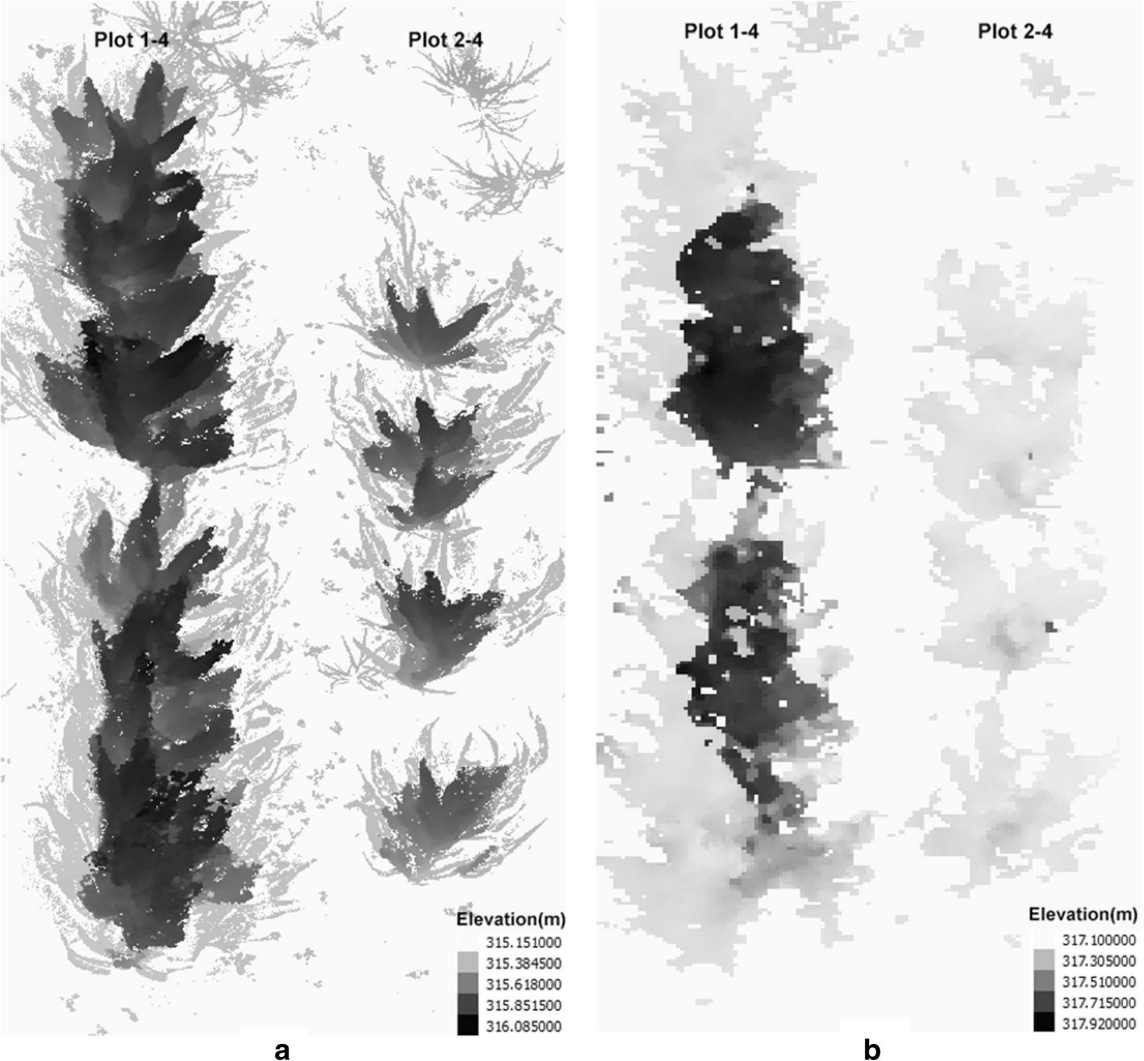
Digital elevation models (DEM) of two plots generated by **a** ground-based proximal imaging by DSLR camera arrays and **b** UAV-based remote imaging by a digital camera

In this study, around 150 high-resolution images captured on the ground were averagely used to generate the proximal DEM of each of the two plots in the same range, while 135 aerial images were used to generate the remote DEM of the entire 80 selected plots. Accordingly, DEMs generated by proximal imaging were visualized much sharper than DEMs generated by remote imaging (Fig. 9). The ground pixel sizes of the DEMs by proximal and remote imaging were 0.2 and 2 cm/pix, respectively. Although there were minor absolute elevation differences between the proximal and remote DEMs, which was due to being georeferenced by two GNSS systems, the plant height would not be affected as it was calculated from the difference between the canopy and the soil elevations. We observed that within the sparsely vegetative plot the plant height by remote imaging was lower than that by proximal imaging. It was likely because remote image acquisition resulted in fewer common key-points among the image overlapping areas within a sparsely vegetative plot for generating the DEM of the canopy. Therefore, the DEM could not delineate a complete canopy profile, resulting in many lower plot-level height values than the manually measured height measurements. In addition, compared with one 12 MP digital camera carried by a UAV, four 18 MP DSLR cameras carried by a ground vehicle for proximal image acquisition provided higher resolution ground images than aerial images and more images in a unit area for DEM generation. Therefore, the 3D canopy profile by proximal imaging resulted in more precision than by remote imaging for plant height extraction. In this study, we did not use as many cameras as Nguyen et al. [13] (i.e., 16 cameras) and did not angle the cameras on the ground vehicle because we only wanted to collect sufficient images for plant height extraction rather than for 3D reconstruction of the complete plant.

### Comparisons of plant height

**Fig. 10:**
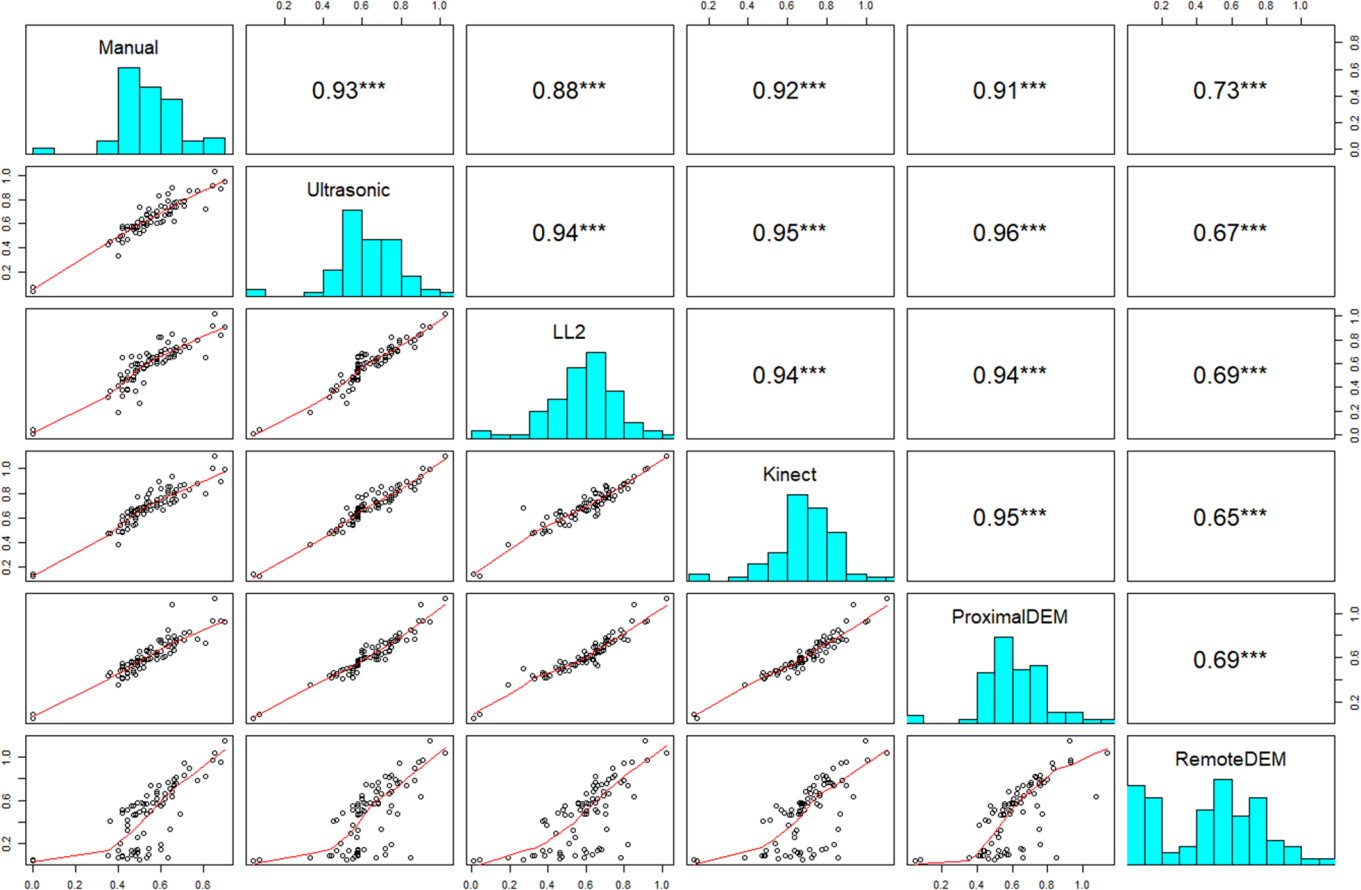
Maximum measurements as plot-level plant height compared with manual measurements. The panels above the diagonal of each figure correspond to the Pearson’s correlation coefficients of plot-level height measurements between each two sensing technologies. The asterisks indicate the level of significance (***P < 0.001). The panels on the diagonal of each figure show the histograms of the 80 plot-level height measurements by each sensing technique. The panels below the diagonal of each figure show the bivariate scatter plots with the spline fit lines

**Fig. 11:**
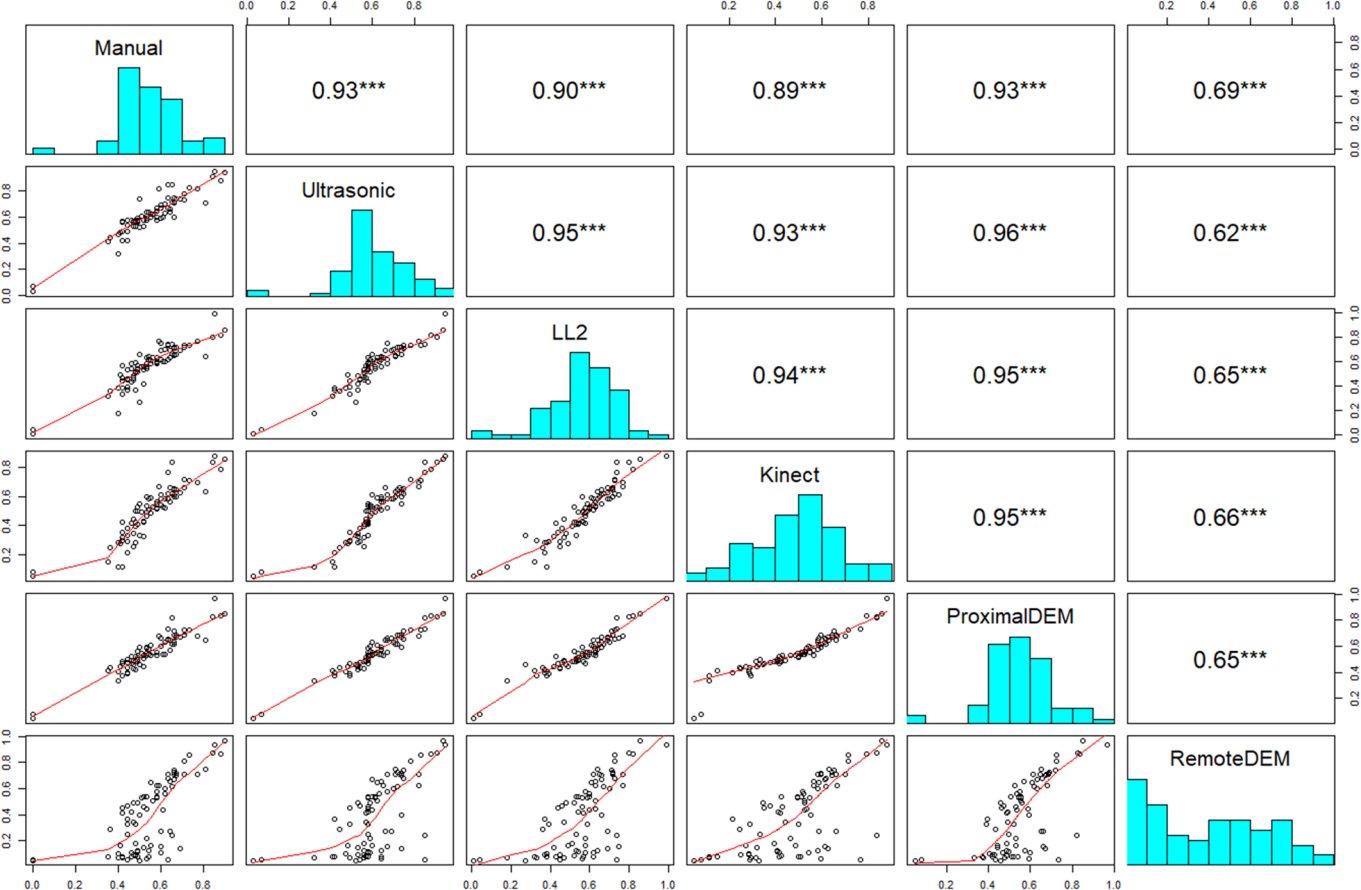
Averaged top 5% measurements as plot-level plant height compared with manual measurements. The panels above the diagonal of each figure correspond to the Pearson’s correlation coefficients of plot-level height measurements between each two sensing technologies. The asterisks indicate the level of significance (***P < 0.001). The panels on the diagonal of each figure show the histograms of the 80 plot-level height measurements by each sensing technique. The panels below the diagonal of each figure show the bivariate scatter plots with the spline fit lines

The plot-level plant height results were derived by both the maximum and the averaged top 5% of the measurements. These two types of plot-level measurements of the 80 selected plots for each of the sensing technologies were compared with manual measurements (Figs. 10, 11).

**Fig. 12:**
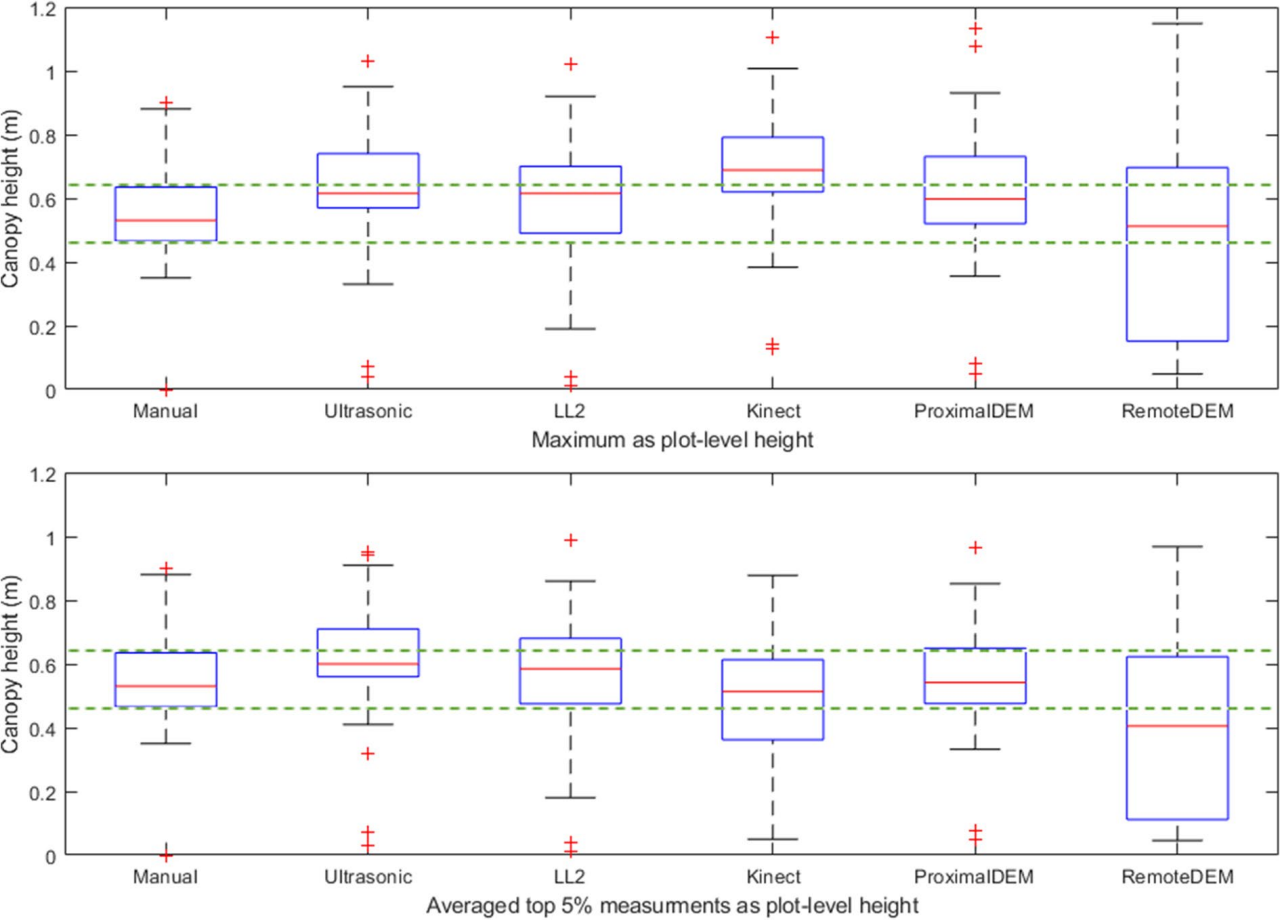
Box plots of plant height measurements of 80 selected plots by different sensing technologies. The upper one used maximum height as the plot-level height, while the lower one used averaged top 5% measurements as the plot-level height

According to the quantitative comparison results (Figs. 11, 12), the plot-level height values measured by the averaged top 5% of the proximal DEM values were the closest to the manual measurements. This is likely due to the ultra-high spatial resolution of the proximal DEM and the relatively larger point sampling size for average height calculation.

**Fig. 13:**
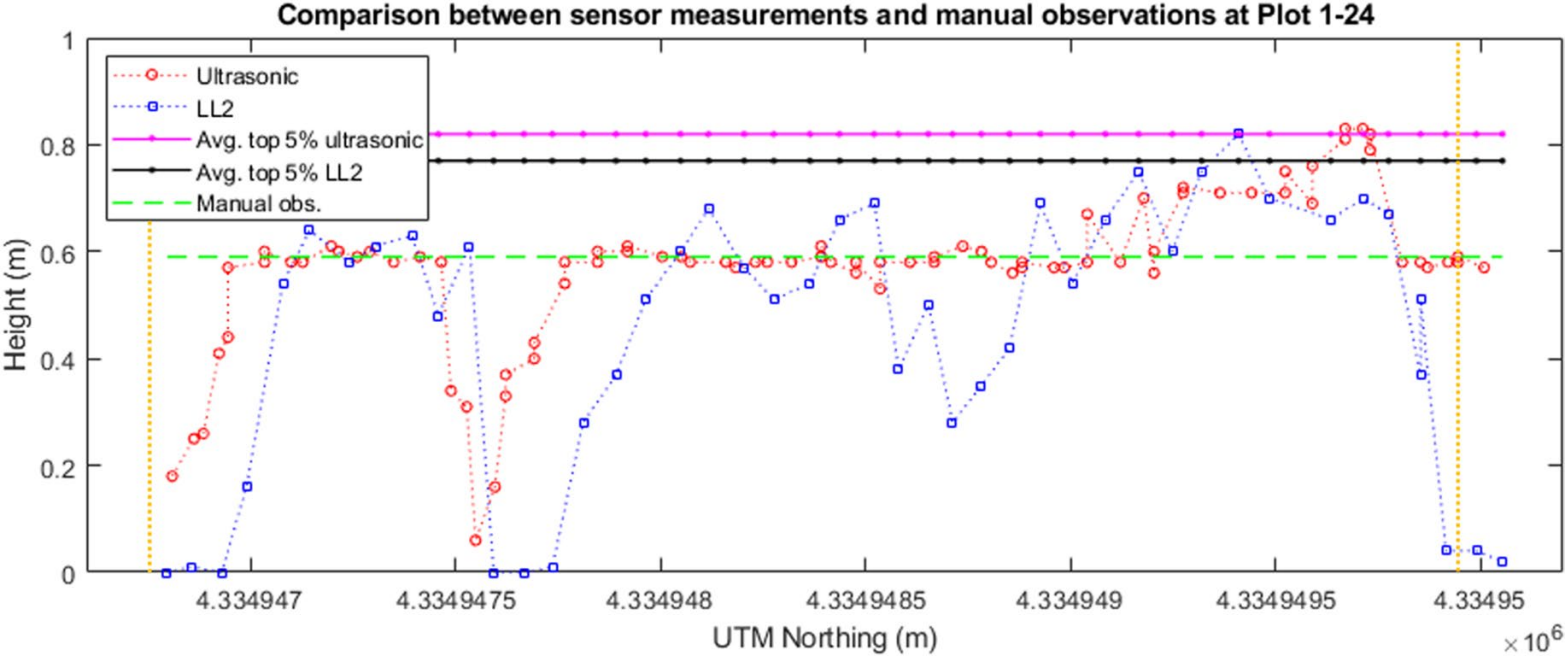
Height observations by the ultrasonic sensor and the LL2 sensor at Plots 1–24. The red circles indicate the ultrasonic sensor measurements, while the blue squares indicate the LL2 sensor measurements. The solid pink and black lines indicate the averaged top 5% observations by the ultrasonic sensor and the LIDAR-Lite v2 sensor respectively. The dashed green line shows the manual (ground truth) observation. The vertical yellow lines mark the start and end of the plot. The *X*-axis in each panel indicate the northing coordinates in meters in the UTM 14 N coordinate system. The *Y*-axis in each panel indicate the distance measurements in meters

The second most accurate plant height measurement method benchmarked to the manual measurements in this study was using the ultrasonic sensor (Figs. 10, 11, 12). We found that using the ultrasonic sensor, the plot-level height calculated either from the maximum measurement or the averaged top 5% of the measurements did not show significant differences. We also observed that the plot-level height measured by the ultrasonic sensor was consistently higher than the manual measurement. A possible reason is that the approach to calculating the plot-level height measured by the ultrasonic sensor is capturing the very highest canopy height from the large field of view; however, this part of the canopy may not reflect the height of the majority part of the plot due to the inner-plot height variance (Fig. 13). The number of measurements within each plot were also much lower for the ultrasonic sensor, giving more sampling error. This issue with the ultrasonic sensor indicates the advantage of the proximal DEM, as the huge sample size of the DEM provides a sufficient representative sample for very accurate averaging.

**Fig. 14:**
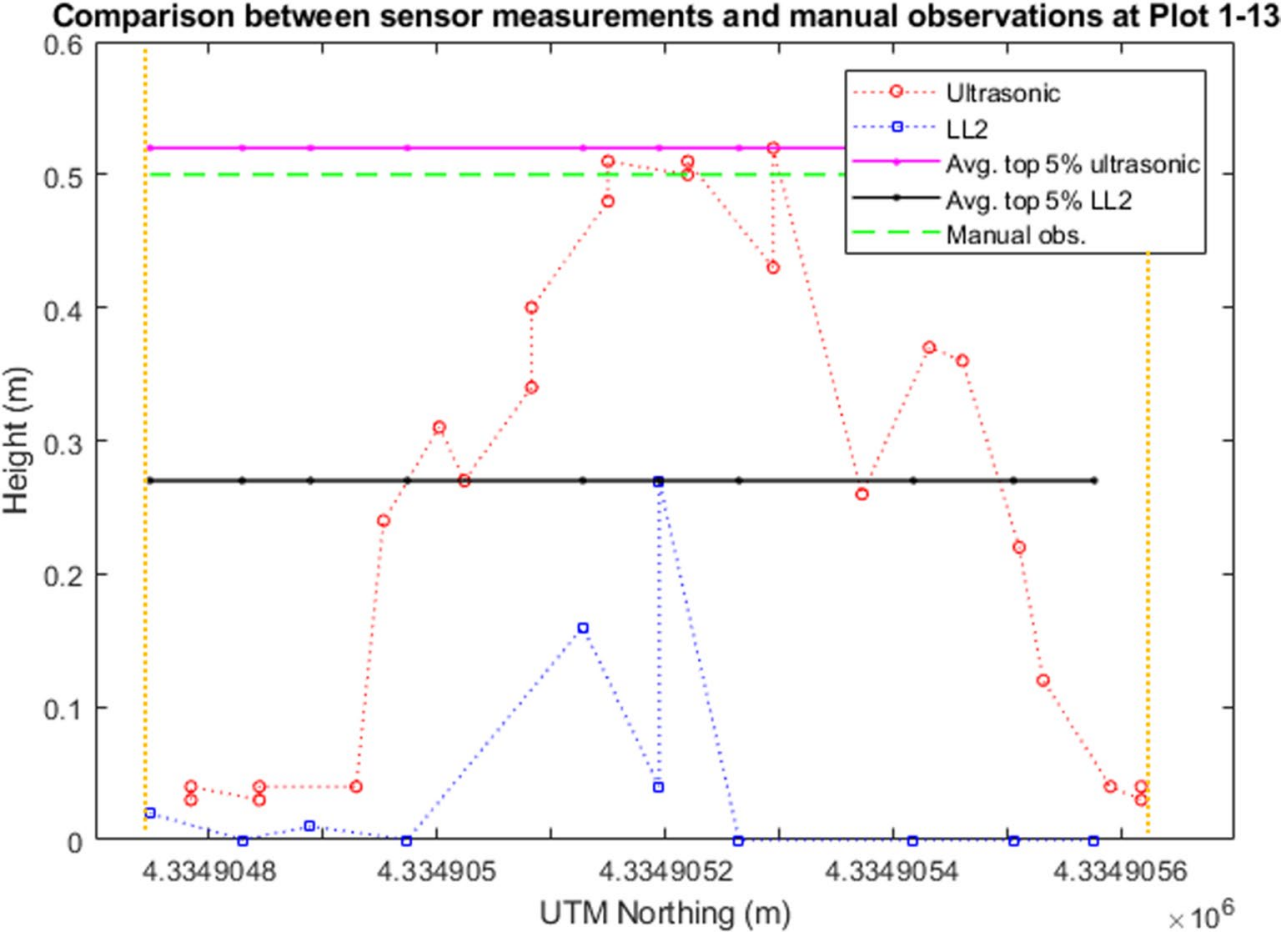
Height observations by the LL2 sensor and the ultrasonic sensor at Plots 1–13. The red circles indicate the ultrasonic sensor measurements, while the blue squares indicate the LL2 sensor measurements. The solid pink and black lines indicate the averaged top 5% observations by the ultrasonic sensor and the LIDAR-Lite v2 sensor respectively. The dashed green line shows the manual (ground truth) observation. The vertical yellow lines mark the start and end of the plot. The *X*-axis in each panel indicate the northing coordinates in meters in the UTM 14 N coordinate system. The *Y*-axis in each panel indicate the distance measurements in meters

The low-cost LL2 sensor has the third highest correlation coefficient value (Figs. 10, 11, 12). A possible reason causing the height measurement differences from the manual measurements was due to the low sampling rate and the pointer-based, narrow FoV of the LL2 sensor (Fig. 14). According to the results provided by the LL2 sensor, there is a reasonable hypothesis that if using a LiDAR, which has a high sampling rate and the capacity to generate a 3D dense point cloud of the canopy, the height measurement accuracy should be highly improved. However, the cost and complexity of these LiDAR systems are likely to be restrictive.

We observed the plot-level plant height using the maximum measurement by the Kinect camera had a higher correlation with the manual measurement than using the averaged top 5% measurements (Figs. 10, 11), but the height values were higher than the manual measurements (Fig. 12). The plot-level height using the averaged top 5% measurements had a lower correlation with the manual measurement, mainly because of the measurements from the plots whose plot-level height were lower than 0.5 m (Fig. 11). According to the observations, it is very likely the Kinect camera has an optimal range for measuring distance. In this study, the canopy located from 0.8 to 1.1 m to the Kinect camera had a better correlation with the manual measurement (Fig. 11). Therefore, if the Kinect camera is mounted at a consistent height above ground level while measuring the plant height, it may not be able to provide accurate results for those canopy out of the camera’s optimal measuring range.

The DEM generated by remote UAV imaging underestimated many plant height measurements (Fig. 12), with many plot-level height values lower than the manual measurements (Figs. 10, 11). In this study, poor emergence due to early-season chilling stress resulted in 34 sparsely vegetative plots among the 80 selected sorghum plots. Considering the limitations of DEMs generated by proximal imaging noted in the previous section, we can partially explain the reason for the low correlation with the manual measurements. We examined the plant height of Plots 1–17, which provided the maximum height difference between the manual measurement and the height measurement by remote imaging. Even the maximum DEM value was used to calculate the plant height, the calculated plant height was still lower than the manual measurement. Therefore, to collect sufficient data to delineate the canopy profiles similar to the field condition in this study, a potential solution is to use a camera with higher resolution (greater than 12 MP) and a lower flight elevation (lower than 25 m).

### Performance comparison

According to the results of this study, we compared the performance of each sensing technology, as shown in Table 3.

**Table 3:** Performance comparison of each sensing technique

Sensors	Carriers	Performance			
		Resolution	Equipment cost per unit	Accuracy compared with the ground truth	Data processing cost
LIDAR-Lite v2 sensor	Ground vehicle	Low, only reflect 1-dimensional measurements	< $100	Low, due to the low sampling frequency	Low
Ultrasonic sensor			$300–$800	High	
Kinect camera		~ 0.2 MP	< $300	High, within the optimal measuring range	
DSLR cameras		~ 18 MP	> $800	Highest	High, due to photogrammetry processing
Digital cameras	UAV	~ 12 MP	< $600	Lowest	

The rank could change with improved data collection settings and data processing approaches. Regarding the resolution, although the digital camera used by remote imaging had a relatively high resolution, if the DEM generated by the aerial images cannot delineate a complete canopy profile, the high camera resolution cannot provide an accurate plot-level height. The rank of the equipment cost only considered the sensing device cost, not including the platform cost. The most time-consuming data processing procedure was generating the DEMs by photogrammetry. As the proximal imaging captured the most high-resolution images, it took the longest time for data processing.

According to what we learned from this study if the plant height accuracy has the priority, an ideal methodology is to use a sensor fusion technology by a LiDAR and an array of high-resolution cameras carried by a ground vehicle platform. The ortho-photo stitched by high-resolution images of the cameras can be registered to the 3D dense point cloud generated by the LiDAR data. Pixel information can be used to extract the plant canopy area. Then the plant height can be calculated from the non-averaged point observations within the canopy area. However, the data collection and data processing are expected to be time-consuming as well as a high equipment cost. If we pursue the low equipment cost and the high calculation efficiency, an array of ultrasonic sensors carried by a ground vehicle platform is an optimal solution for the plant height measurement, but using this method may still lose geospatial resolution compared with image data. If the data collection efficiency is with more concern, such as data collection in a large-scale breeding field, using a LiDAR and a high-resolution camera carried a UAV is a feasible solution; however, there will be huge cost from the equipment and the data processing.

## Conclusions

We investigated five different sensing technologies for field-based HTPP of plant height with a case study in sorghum. Using the data collection approaches and the data processing methods introduced in this study, we found the plot-level height values measured by the ultrasonic sensor, the LL2 sensor, the Kinect camera, and the proximal imaging by four DSLR cameras were all highly correlated with the manual measurements. Therefore, each sensing technique could be used for precisely and quickly measuring large numbers of sorghum genotypes to identify height variance.

Although the height measured by remote imaging had a lower correlation with the manual measurements in this study, the accuracy can be improved by using a higher resolution camera and collecting the images at a lower flight altitude. In fact, HTPP by remote sensing with UAV has vast potential due to its fast data collection speed, compatibility to plants with different morphological traits, and high geospatial resolution. Subsequent studies on UAV phenotyping will focus on tuning the data collection settings and refining the plant trait extraction algorithms.

We provided data processing method for each sensing technology to extract the plot-level plant height. These methods can be of immense value in HTPP of diverse germplasm subjected to different biotic and abiotic stressors that cause reduced crop stand and impacting plant height. We found deriving the plot-level height by averaging a portion of the highest sensing observations would work for densely vegetative plots, but not for those sparsely vegetative plots. Future studies will focus on the fusion of the sensor measurements (i.e., the LiDAR point cloud) and pixels information to build ultra-high resolution plant structure models for complex trait extraction. Also, as the sensing approaches of using the Kinect camera and DSLR imaging arrays provide both color and morphological information, they can assist in identifying plants, quantifying the plant counts, and measurement of individual plants in those sparsely vegetative plots. In conclusion, the approaches investigated in this study for HTPP of sorghum height can be applied to measure plant height of any number of other crops, such as corn, cotton, and soybeans, and enable more precise and higher throughput measurements for breeding, genetics, and agronomic studies.

## Abbreviations

2D: 2-dimensional
3D: 3-dimensional
BGNir: blue-green-near infrared
DEM: digital elevation model
DSLR: digital single-lens reflex
FoV: field of view
GCP: ground control point
GNDVI: green normalized difference vegetation index
HIS: hue-saturation-intensity
HTPP: high throughput plant phenotyping
KSU: Kansas State University
LiDAR: light detection and ranging
LL2: LIDAR-Lite v2
MP: mega pixels
PheMU: phenotyping mobile unit
QGIS: quantum geographical information system
RGB: red–green–blue
RIL: recombinant inbred line
SfM: structure-from-motion
ToF: time-of-flight
UAS: unmanned aerial system
UAV: unmanned aerial vehicle
UTM: universal transverse mercator
